# A Cost-effective Phenothiazine-based Fluorescent Chemosensor for Selective Detection of Hydrazine and its Application To Real Water Samples

**DOI:** 10.1007/s10895-025-04421-9

**Published:** 2025-06-25

**Authors:** Özge Çağlar Teknikel, Nebahat Değirmenbaşı

**Affiliations:** 1 Graduate School of Natural and Applied Sciences, Gazi University, Ankara, 06500 Türkiye; 2 Faculty of Science, Department of Chemistry, Gazi University, Ankara, 06500 Türkiye

**Keywords:** Phenothiazine, Hydrazine, Fluorescence, Turn on sensor, Selectivity, Cost-effective

## Abstract

Hydrazine is a highly toxic yet industrially indispensable compound extensively employed in aerospace, pharmaceutical, and polymer sectors. Its pronounced carcinogenicity and ecological persistence necessitate the development of sensitive and selective detection strategies, particularly for aqueous environments. Herein, we present **PHENOZ**, a cost-effective and structurally simple phenothiazine-based fluorescent probe functionalized with a dicyanovinyl moiety, capable of selectively detecting hydrazine with a prominent turn-on fluorescence response. **PHENOZ** was synthesized in only two steps from commercially available starting materials. The probe displayed no background fluorescence in the absence of hydrazine; however, a pronounced emission emerged upon hydrazine addition. This behavior is attributed to a nucleophilic attack of hydrazine on the electron-deficient dicyanovinyl group, which significantly alters the electronic structure of the molecule and activates the fluorescence pathway. A strong linear correlation between fluorescence intensity and hydrazine concentration was observed in the 0–40 µM range, with a detection limit of 0.22 µM (7.05 ppb) —well below the ACGIH toxicity threshold for aquatic systems. Selectivity experiments confirmed that **PHENOZ** exhibited no significant response toward potentially interfering species such as cyanide and primary amines. Furthermore, real sample analyses in tap, bottled, and lake water demonstrated recovery rates ranging from 94.7 to 111.1%, confirming the probe’s potential for practical environmental monitoring. Taken together, these results establish **PHENOZ** as a highly selective, simple, and effective fluorescent chemosensor for hydrazine detection in aqueous media.

**Supplementary Information:**

The online version contains supplementary material available at 10.1007/s10895-025-04421-9.

## Introduction

Hydrazine, valued for its versatile chemical properties, plays a crucial role across a wide range of industrial, technological, and pharmaceutical applications. Owing to its high energy content and ease of combustion, hydrazine is widely utilized as rocket fuel [1, 2], prominently featuring in space vehicles [1–3] and military applications [4]. Hydrazine finds extensive use in the polymer industry [1, 5, 6]. Specifically, it serves as a radical initiator in polymerization processes [7], facilitates the synthesis and production of conducting polymers such as polyaniline [8], acts as a catalyst in the formation of high-performance polymers like polyimides [9, 10], which are characterized by their thermal stability and mechanical strength, and is also employed as a cross-linking agent [11]. Hydrazine holds significant importance in the pharmaceutical industry, with several crucial therapeutic agents being synthesized using this compound [12–15]. Notably, isoniazid, an antitubercular drug, and hydralazine, an antihypertensive agent, are among the pharmaceuticals produced through hydrazine-based synthesis [16, 17]. These examples illustrate the essential role of hydrazine in the development of effective treatments for various medical conditions. Despite its wide range of usage and applications, hydrazine poses a significant source of risk for both environment and human health. Hydrazine is a highly toxic and reactive compound, requiring strict exposure controls because of its considerable risks [18–21]. The Occupational Safety and Health Administration (OSHA) [22], the National Institute for Occupational Safety and Health (NIOSH) [18], the American Conference of Governmental Industrial Hygienists (ACGIH) [22], and other regulatory agencies have set very low permissible exposure limits for hydrazine due to its carcinogenicity, genotoxicity, and high reactivity. For instance, the Threshold Limit Value (TLV) recommended by the American Conference of Governmental Industrial Hygienists (ACGIH) is 0.31 µM (10 ppb), reflecting the compound’s severe toxicity even at trace levels [23]. Similarly, the US-EPA and IARC have emphasized its hazardous nature, urging strict regulation and continuous monitoring. Consequently, the sensitive detection of hydrazine in the environment is crucial for ensuring occupational safety and ecological protection.

Studies presented in the literature have demonstrated that hydrazine exposure can lead to various acute health problems [19, 24–26]. These health issues include irritation, pulmonary edema and pleural effusions [18], central nervous system disorders [27], depression [18] and cell necrosis [28]. Prolonged exposure above established limits can result in kidney and liver damage [28], hematological disorders [18, 28, 29], and an increased risk of cancer [20, 26]. Given all these reasons, the detection and quantification of hydrazine becomes critically important [30]. Various traditional analytical methods, including electrochemical analysis [31, 32], capillary electrophoresis [17], chemiluminescent techniques [33], colorimetrical methods [34], chemical titration [35], and chromatography [36] have been employed for detection of hydrazine. Another technique that has proven itself apart from these methods is fluorescence spectroscopy. It has proven highly advantageous due to its low cost, high selectivity, real-time detection capability, specificity and time efficiency [37–45].

In recent years, various heterocyclic compounds have been extensively explored as fluorescent chemosensors for the selective detection of hydrazine, owing to their tunable electronic properties and strong fluorescent responses. For instance, triazole-functionalized fluorophores have demonstrated off–on type fluorescence responses upon nucleophilic addition of hydrazine, offering both sensitivity and selectivity in aqueous media [46]. Similarly, benzothiazole-based systems have been reported to exhibit strong photophysical shifts upon hydrazine binding, attributed to effective conjugation and electron-deficient recognition sites [47]. Quinolines have also emerged as efficient substances for hydrazine sensing, often utilizing intramolecular charge transfer (ICT) mechanisms to achieve ratiometric or turn-on fluorescence behavior [48]. Additionally, rhodamine-based systems have attracted attention due to their colorimetric and fluorescent dual-sensing abilities, particularly in biological and aqueous environments [49]. Coumarin and dansyl derivatives have also demonstrated promising performance as turn-on sensors in live-cell imaging and environmental monitoring [50, 51]. Furthermore, β-dicyanovinyl-substituted calix[4]pyrrole derivatives have shown excellent performance as colorimetric and fluorometric probes, benefiting from electron-deficient recognition sites and rigid macrocyclic frameworks [52]. BODIPY [53], pyrene [54, 55], phenothiazine [56] and squaraine [57] are the other cores that also have been employed for the detection of hydrazine. These studies collectively highlight the versatility of heterocyclic scaffolds in chemosensor design and reinforce the relevance of phenothiazine-based hydrazine sensors, which offer robust fluorescence, excellent photostability, and tunable π-conjugated systems, making them well-suited for selective hydrazine detection through turn-on or turn-off fluorescence mechanisms.

Phenothiazine is a heterocyclic compound with a tricyclic structure that includes two benzene rings and a central thiazine ring. Its exceptional photostability, strong fluorescence, and high quantum yields make it highly effective for sensor applications [56, 58–63]. The compound’s significant electron-donating properties enhance its interaction with analytes. The sensors maintain performance over time due to phenothiazine’s robustness under light exposure [64]. Its versatility allows for custom modifications to tailor sensors for various applications. Overall, phenothiazine’s attributes make it an ideal choice for developing reliable fluorescent sensors for various analytical needs [65–67].

Fluorescent hydrazine chemosensors typically require multi-step synthesis pathways resulting in higher costs due to the necessity of expensive materials [68–71]. The dicyanovinyl group is frequently used as a recognition unit for hydrazine in these chemosensors. The electron-deficient carbon atom within this group reacts with various nucleophilic species (hydrazine, amines, cyanide), enabling its use for the detection of such analytes [63, 72–74]. However, the reaction of different nucleophiles with this group limits its application as a selective sensor. To the best of our knowledge, no hydrazine chemosensor has been reported to be unreactive towards both cyanide and amine. In this study, we report our findings on a phenothiazine compound functionalized with a dicyanovinyl group, which exhibits totally selective fluorescent responses to hydrazine ions. The selectivity studies revealed that this chemosensor is non-responsive to even highly potentially responsive species such as cyanide and primary amines [72–74].

## Experimental

### Material and Instrumentation

Phenothiazine, malononitrile, hydrazine hydrate, and other reagents were supplied commercially. They were purchased from Sigma-Aldrich and used in the synthesis without further purification. All inorganic salts used in the selective detection of hydrazine were purchased from Sigma-Aldrich with analytical grade purity and were used without further processing. The characterization of all compounds was performed using high-resolution mass spectrometry, ^1^H NMR, ^13^C NMR, and FTIR-ATR techniques. The mass spectra of the compounds were recorded on an Agilent Technologies 6530 Quadrupole Time-of-Flight (Q-TOF) LC/MS. The NMR spectra of the compounds were recorded on a Bruker Avance Neo 500 NMR spectrometer. FTIR-ATR spectra were recorded using a Thermo Scientific Nicolet IS5 FT-IR Spectrophotometer. The reaction progress was monitored using thin layer chromatography (Kieselgel 60, F254, E. Merck). The products were purified through column chromatography, employing silica gel (0.05–0.063 nm, 230–400 mesh, ASTM, Merck) as the stationary phase. Fluorescence spectra were obtained with a Thermo Scientific Lumina spectrofluorometer, with both emission and excitation slit widths set to 5 nm. Boric acid buffer was employed to perform measurements within the pH range 4.0 to 10.0.

### Syntheses

#### Synthesis of 1

To formylate phenothiazine literature method [75] was used with some modifications. To the solution of Phenothiazine (1 g, 5 mmol) in acetic acid (10 mL) was added hexamethylenetetramine (HMTA) (0.8 g, 5.5 mmol) and the reaction mixture was refluxed for 24 h. After the completion of the reaction (TLC), the reaction mixture was cooled to room temperature and poured into saturated sodium bicarbonate solution in an ice bath. The reactant was extracted with ethyl acetate. Organic phase was dried by using Na_2_SO_4_. Na_2_SO_4_ was filtrated and the crude product was purified by column chromatography (1:4 ethyl acetate: hexane). Powdery orangish yellow solid (2.76 mmol, 0.628 g, 55%). mp = 175 °C. FT-IR (cm^− 1^): 3336, 3050, 2918, 2834, 2751,1654,1595,1558, 1465,1433, 1189. ^1^H NMR (500 MHz, DMSO) δ 9.64 (s, 1H), 9.26 (s, 1H), 7.51 (d, *J* = 8.2 Hz, 1H), 7.49 (s, 1H), 7.00 (t, *J* = 7.6 Hz, 1H), 6.99 (d, *J* = 7.6 Hz, 1H), 6.83 (t, *J* = 7.4 Hz, 1H), 6.73-6,62 (m, 2 H). ^13^C NMR (100 MHz, DMSO) δ 190.43, 147.61, 140.05, 130.91, 128.35, 127.88, 126.75, 123.59, 117.15, 116.22, 115.58, 114.44. HRMS (APCI-TOF m/z): Calculated for C_13_H_8_NOS [M-H]^−^ 226.0327; Found 226.0357.

#### Synthesis of PHENOZ

To obtain **PHENOZ** literature method [56] was used with some modifications. Compound **1** (0.405 g, 1 mmol) and malononitrile (0.100 g, 1.5 mmol) were dissolved in EtOH (10 mL), followed by addition of a 2–3 drops of piperidine. Then, the mixture was stirred for 4 h under room temperature. After the completion of the reaction (TLC), the solvent was removed *in vacuo*. The crude powder was washed with diethyl ether to obtain probe **PHENOZ.** Crimson solid (0.490 g, 80%). mp = 202 ^o^C. FT-IR (cm^− 1^): 3318, 2220, 1604, 1581, 1548, 1506, 1471, 1244, 1157. ^1^H NMR (500 MHz, DMSO): δ 9.64 (s, 1H), 8.05 (s, 1H), 7,62 (d, *J* = 8.0 Hz, 1H), 7.44 (s, 1H), 7.02 (t, *J* = 7.2 Hz, 1H), 6.92 (d, *J* = 7.2 Hz, 1H), 6.83 (t, *J* = 7.1 Hz, 1H), 6.72–6.60 (m, 2 H). ^13^C NMR (100 MHz, DMSO) δ 158.73, 147.59, 138.85, 132.88, 128.88, 128.48, 126.75, 125.26, 124.17, 117.36, 116.09, 116.05, 115.80, 114.91, 114.69, 74.13. HRMS (APCI-TOF, m/z): Calculated for C_16_H_8_N_3_S [M-H]^−^ 274.0439; Found 274.0459.

#### Synthesis of PHENOZ-NNH_2_

**PHENOZ** (60 mg, 0.218 mmol) was completely dissolved in 2 mL of DMSO. Then, mixture of EtOH (19 mL)/H_2_O (1 mL) solution was added to the obtained solution. Hydrazine (54 µL) was added to the this mixture, and the color of the solvent changed from magenta to pale yellow at room temperature. The mixture was stirred for 6 h. After the completion of the reaction the solvent was removed *in vacuo*. The crude mixture was washed with iced water and the entire mixture was extracted with ethyl acetate. The organic phase was removed *in vacuo*. The crude product was purified by column chromatography (1:6 ethyl acetate: hexane). Pale yellow solid (30 mg, 58%). FT-IR (cm^− 1^): 3325, 3054, 1680, 1600, 1572, 1506, 1470, 1307, 1272,1202. ^1^H NMR (500 MHz, DMSO): 9.01 (s, 1H), 8.45 (s, 1H), 7.45 (dd, *J* = 8.3, 1.8 Hz, 1H), 7.36 (d, *J* = 1.9 Hz, 1H), 7.01 (td, *J* = 7.7, 1.5 Hz, 1H), 6.93 (dd, *J* = 7.7, 1.5 Hz, 1H), 6.79 (td, *J* = 7.5, 1.2 Hz, 1H), 6.74–6.67 (m, 2 H).^13^C NMR (100 MHz, DMSO) δ 160.22, 144.75, 140.98, 128.90, 128.25, 128.08, 126.74, 126.18, 122.95, 117.14, 116.27, 115.26, 114.77. HRMS (APCI-TOF m/z): Calculated for C_13_H_12_N_3_S [M + H]^+^ 242.0752; Found 242.0766.

### Quantum Yield

The fluorescence quantum yields of the compounds were determined using fluorescein as the reference standard (Φ_fl._ = 0.79 in ethanol) [76]. The relative quantum yield of each sample was calculated according to the following equation:

$$\:{{\Phi\:}}_{fl}={{\Phi\:}}_{r}\frac{{A}_{s}}{{A}_{r}}\:\frac{{I}_{r}}{{I}_{s}}\:\frac{{\eta\:}_{{s}^{2}}}{{\eta\:}_{{r}^{2}}}$$

where Φ_fl._ and Φ_r_ represent the quantum yields of the sample and the reference, respectively. $\:A$ is the absorbance at the excitation wavelength. *I* is the integrated emission intensity; and *η* is the refractive index of the solvent. In this study, both the sample and the reference were dissolved in ethanol, ensuring consistent solvent environments. Based on this approach, the fluorescence quantum yield of **PHENOZ-NNH**_**2**_ was determined to be 0.05 in EtOH.

## Results and Discussion

### Synthesis of PHENOZ

The synthesis of **PHENOZ** (Scheme 1), consisting of a phenothiazine core and a dicyanovinyl group, was achieved via the synthetic pathway illustrated in Scheme 1. In the first step, the phenothiazine core was formylated at the 3-position through the Duff reaction to yield compound **1**. Subsequently, compound **1** was heated with malononitrile in the presence of a piperidine catalyst to produce **PHENOZ**. The synthesized compounds were characterized using ¹H NMR, ¹³C NMR, FTIR, and HRMS techniques (Figs. S1-S15).

**Scheme 1:**
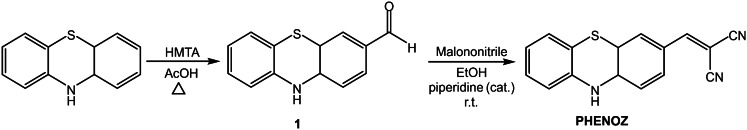
Synthetic route for the synthesis of **PHENOZ**

### Photophysical Investigation of PHENOZ

The photophysical properties of the **PHENOZ** were investigated in EtOH: H₂O (9:1). The compound exhibited the maximum absorption band at 484 nm (Fig. S16). When excited at this wavelength, no emission band was observed. The quenching of fluorescence is quite expected for such a compound because of the intramolecular charge transfer (ICT) occurring from the electron-donating phenothiazine core to the electron-deficient dicyanovinyl unit in the excited state. Upon the addition of hydrazine, a pronounced turn-on fluorescence response was observed, indicating a strong interaction between hydrazine and the dicyanovinyl unit. Among the tested solvent systems the best results were observed to be obtained in 9:1 EtOH: H_2_O system. Therefore, 9:1 EtOH: H_2_O was chosen as the solvent mixture.

### Selectivity and Comperative Experiments

In the next stage, it was aimed to investigate the photophysical responses of **PHENOZ** to various species. Given that the dicyanovinyl group is commonly used in the literature for the fluorimetric detection of cyanide anions [45, 62, 63], we especially included these test CN⁻ along with F⁻, Cl⁻, I⁻, NO₃⁻, HSO_3_^−^, HSO₄⁻, H₂PO₄⁻ and ClO^−^ anions. For neutral species, we focused on those likely to generate a fluorescence response with the **PHENOZ** through a nucleophilic attack to electron-deficient dicyano carbon. These species include ammonia, pentylamine, morpholine, triethylamine, aniline, phenol, cysteine, glutathione and thiourea. The cations tested included Cu²⁺, Ni²⁺, Mg²⁺, Ag²⁺, Hg²⁺, Cd²⁺, Pb²⁺, Zn²⁺, and Fe³⁺.

**Fig. 1:**
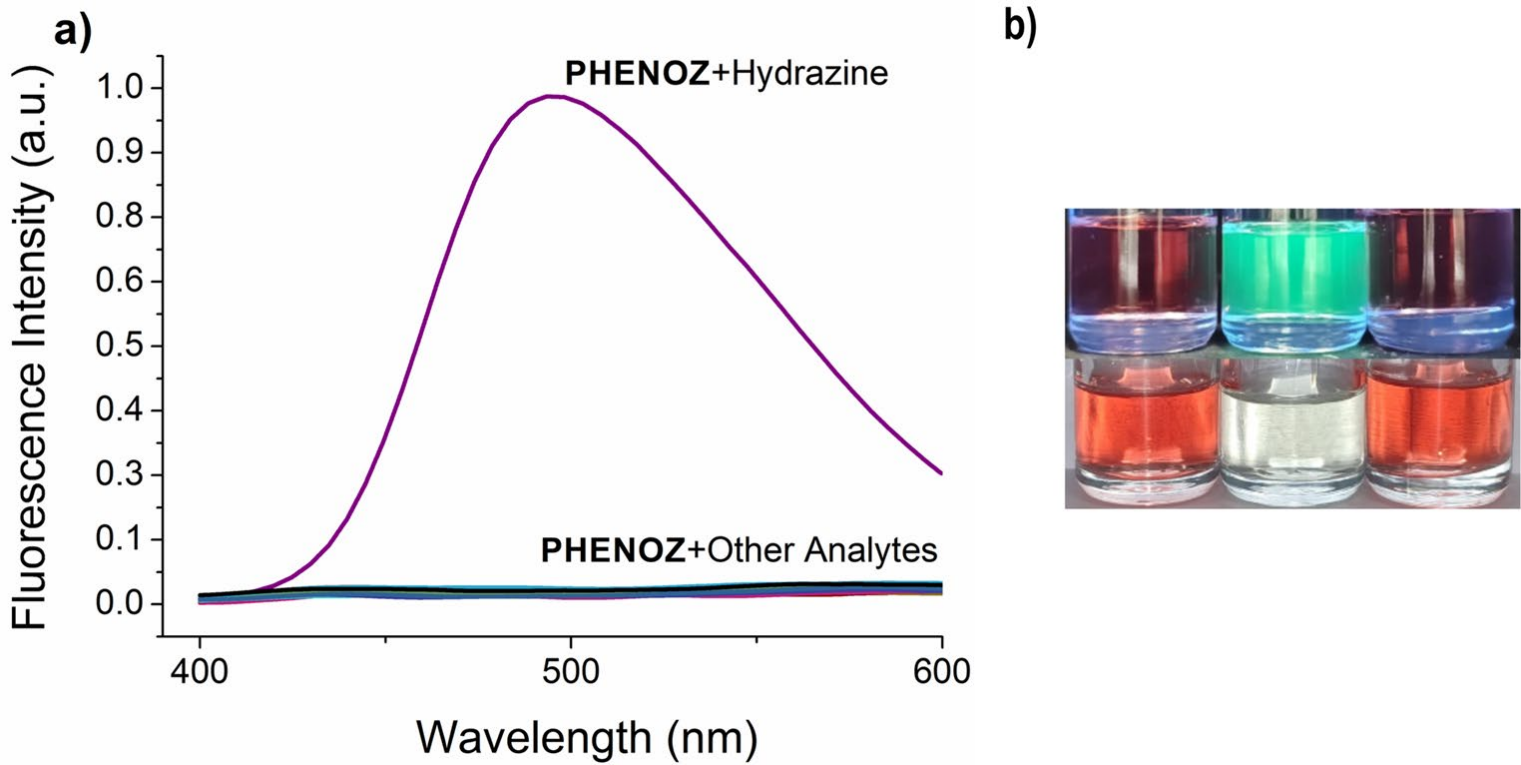
**a**) The fluorescence emission spectra of sensor **PHENOZ** (50 µM) solutions upon addition of different species (15 µM). **b**) Images of the solutions containing **PHENOZ** (50µM), **PHENOZ**–Hydrazine (50 + 15 µM), and **PHENOZ**–Pentylamine (50 + 15 µM) were obtained under excitation at different wavelengths. The species expected to exhibit the highest level of interference was selected as a representative.(Top: Under 254 nm, bottom: under sunlight; left: **PHENOZ**, middle: **PHENOZ** + hydrazine, right: **PHENOZ** + pentylamine, respectively)

**Fig. 2:**
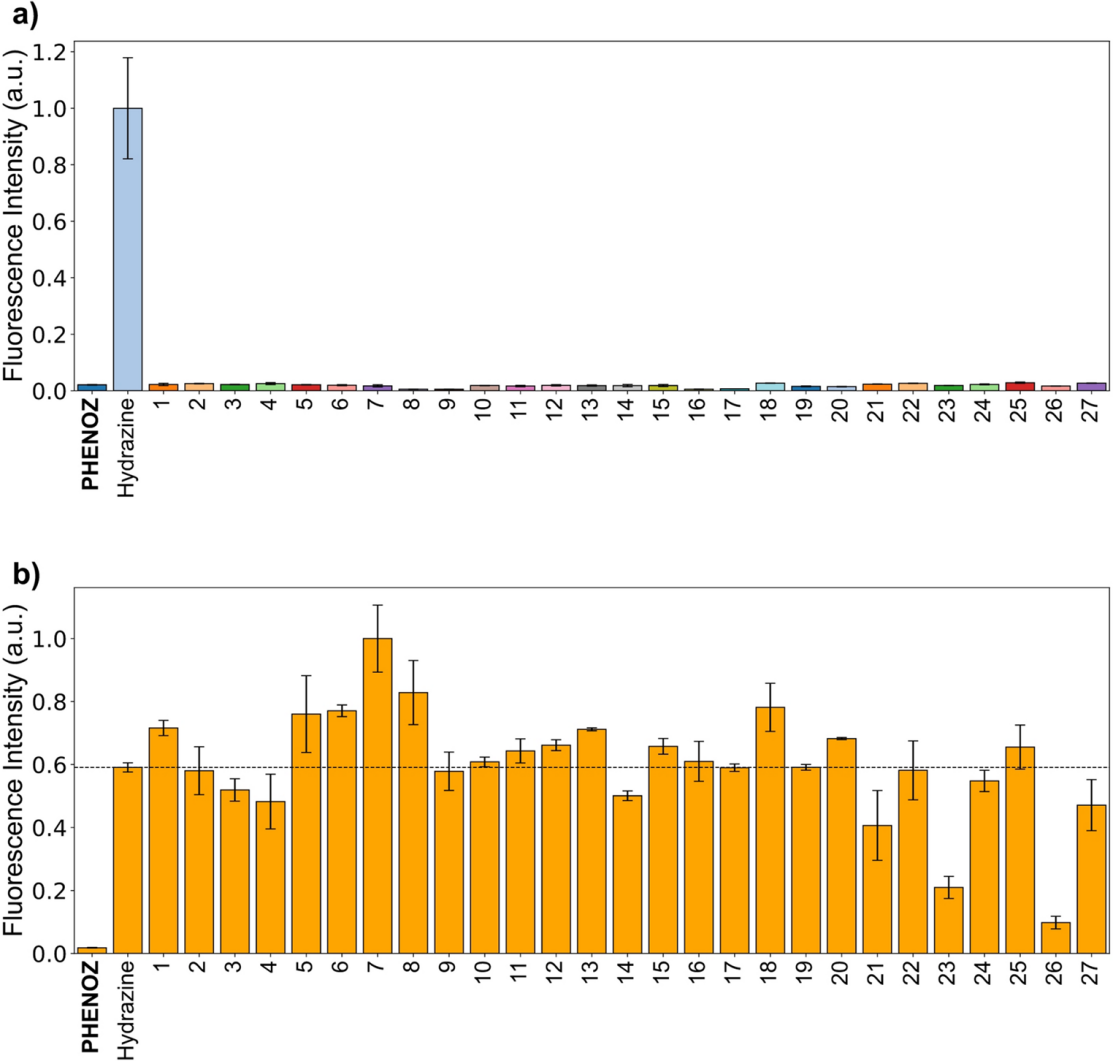
**(a)** The fluorescence emission spectra column graph of sensor **PHENOZ** (50 µM) solutions upon addition of different species (15 µM). **(b)** The fluorescence emission spectra of **PHENOZ** (50 µM) + hydrazine (15 µM) + different species (15 µM). (1: Phenol, 2: Aniline, 3: Morpholine, 4: TEA, 5: Ammonia, 6: Thiourea, 7: Penthylamine, 8: Cysteine, 9: Glutathione, 10: NO_3_^⁻^, 11: Cl^⁻^, 12: I^⁻^, 13: F^⁻^, 14: HSO_4_^⁻^, 15: HSO_3_^⁻^, 16: H_2_PO_4_^⁻^, 17: ClO^⁻^, 18: CN^⁻^, 19: Hg²⁺, 20: Fe³⁺, 21: Pb²⁺, 22: Zn²⁺, 23: Cu²⁺, 24: Ni²⁺, 25: Mg²⁺, 26: Ag^2^⁺, 27: Cd²⁺)

To perform the photophysical response tests, a 50 µM solution of **PHENOZ** was prepared in EtOH: H₂O (9:1). To each solution, mentioned species were added to achieve a final concentration of 15 µM. Upon the addition of hydrazine, the pinkish color of the solution turned into a colorless, while no color change was observed in the other solutions. Upon the addition of hydrazine the absorption band of **PHENOZ** disappeared and a new band at 430 nm emerged (Fig. S16). No changes were observed in the absorption spectra of solutions containing the other species. In the solution with hydrazine, a very intense fluorescence band with a maximum at 500 nm was observed (Fig. 1) No new fluorescence band appeared in the solutions containing these species (Fig. 2a). These results showed that **PHENOZ** responded to hydrazine with fluorescence turn-on with a remarkable selectivity.

Specificity is a crucial parameter in evaluating the analytical performance of fluorescent probes. To assess the selectivity and interference resistance of the **PHENOZ** probe, a comprehensive study was conducted.

To assess the interference tolerance of the **PHENOZ** sensor, a series of fluorescence measurements were conducted in ethanol solutions containing 10% water. **PHENOZ** (50 µM) was treated with various potentially interfering neutral and ionic species (15 µM) and allowed to equilibrate for 30 min. Subsequently, hydrazine (15 µM) was added to each solution, and fluorescence measurements were recorded after an additional 15 min. The results demonstrated that common anions frequently found in environmental water sources, including Cl⁻, NO₃⁻, HSO₄⁻, H₂PO₄⁻, and F⁻, did not induce significant interference with the hydrazine response of the probe. Similarly, prevalent cations such as Mg²⁺ and Fe³⁺ exhibited negligible influence on the fluorescence signal (Fig. 2b). Pentylamine caused a substantial fluorescence enhancement of approximately 69%. In contrast, Cu²⁺ and Ag^2^⁺ induced considerable quenching effects, leading to fluorescence decreases of 65% and 83%, respectively. Cyanide (CN⁻) exhibited a minor positive interference under the same conditions. Although Cu²⁺, Ag^2^⁺, and pentylamine are not typically abundant in natural water bodies, their presence in industrial or wastewater samples cannot be ruled out. Therefore, the application of the **PHENOZ** probe for hydrazine detection in such matrices should be approached with caution.

### Titration Experiments

**Fig. 3:**
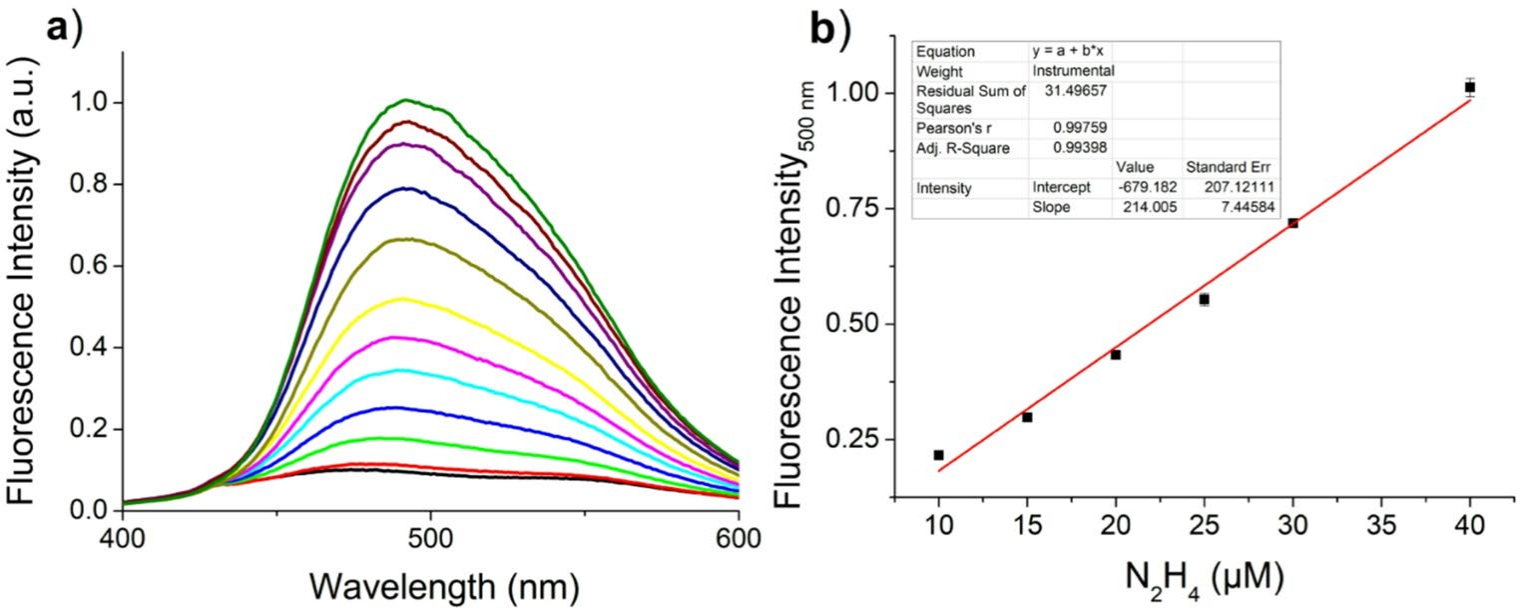
**(a)** Fluorescence spectra of the chemosensor at different N_2_H_4_ concentrations (0–60 µM) **(b)** The linear plot of the fluorescence emission intensity with increasing concentration of hydrazine in the range from 0 to 40 µM

To evaluate the quantitative sensing capability of PHENOZ towards hydrazine, titration experiments were conducted under optimized solvent conditions. The change observed with the addition of hydrazine at increasing concentrations was linear between 0 and 40 µM (Fig. 3b). The correlation coefficient for this graph was calculated as R^2^ = 0.99398. Based on this calibration curve, the limit of detection (LOD) was calculated using the standard formula: LOD = 3σ / S. Where σ is the standard deviation of the blank measurements (*n* = 3) and S is the slope of the calibration curve. The calculated LOD was 0.22 µM. This value is lower than the 0.31 µM toxicity limit set by the American Conference of Governmental Industrial Hygienists (ACGIH) for aquatic environments, confirming the high sensitivity of the sensor. In addition, the limit of quantification (LOQ) was calculated using the formula: LOQ = 10σ / S, resulting in a LOQ value of 0.73 µM (23.4 ppb). This confirms that the sensor can not only detect but also accurately quantify hydrazine at environmentally relevant levels (Fig. 3a, b).

### Determination of pH Working Range

**Fig. 4:**
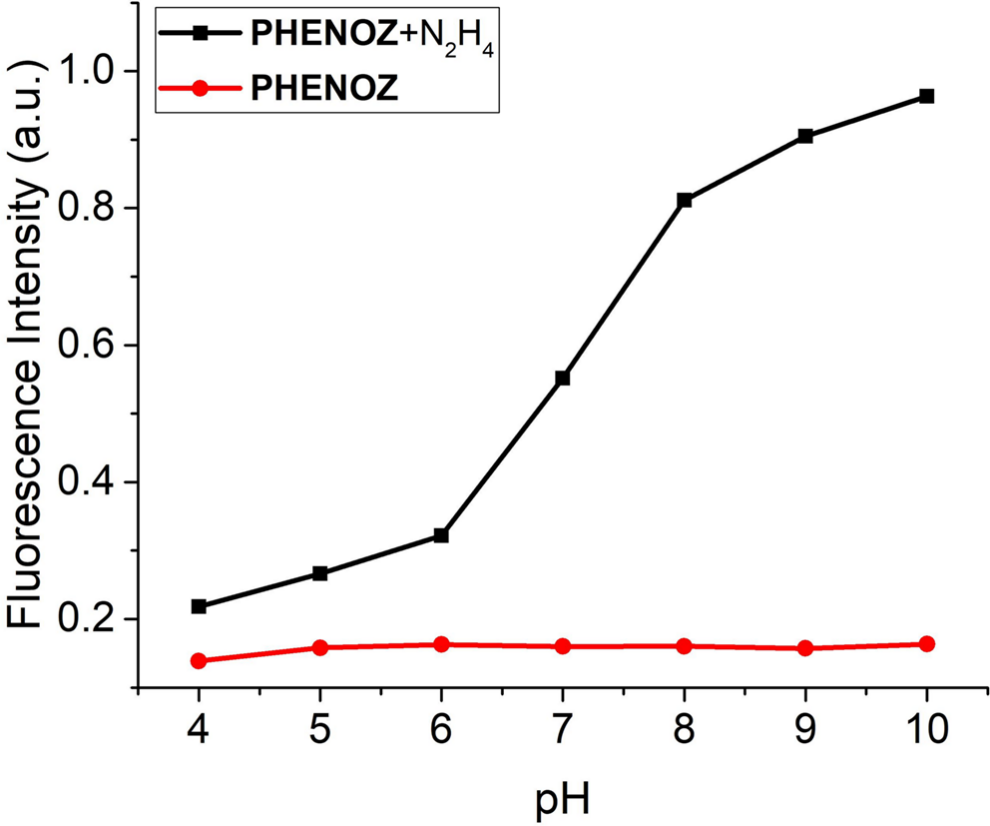
Fluorescence intensity changes of 50 µM PHENOZ in the presence of 15 µM N_2_H_4_

The pH-dependent fluorescence response of **PHENOZ** and its interaction with hydrazine (N₂H₄) was systematically investigated over a pH range 4.0 to 10.0 (Fig. 4). As shown in the figure, **PHENOZ** alone exhibited negligible variation in fluorescence intensity across the entire pH range, indicating high photostability and minimal background signal under both acidic and basic conditions. However, upon addition of hydrazine, a pronounced enhancement in fluorescence intensity was observed, particularly from pH 7.0 onwards. The fluorescence response remained minimal at lower pH values (4.0–6.0), suggesting that the nucleophilicity of hydrazine is significantly suppressed under acidic conditions, likely due to its protonation. In contrast, at near-neutral to basic pH (≥ 7.0), **PHENOZ** demonstrated a substantial and concentration-dependent fluorescence enhancement in the presence of N₂H₄, with the signal reaching its maximum around pH 10. This pronounced pH dependence underscores the importance of protonation–deprotonation equilibrium in modulating the reactivity of hydrazine and confirms that **PHENOZ** functions as an effective hydrazine-responsive fluorescent probe under physiological to mildly alkaline conditions.

### Real Sample Application

Tests were conducted to evaluate the usability of the chemosensor in the analysis of real samples. To evaluate the practical applicability of the system, real water samples comprising tap water, commercially available bottled water, and surface water collected from Lake Eymir (Ankara, Turkey) were fortified with predetermined concentrations of hydrazine. Then the concentrations of these solutions were measured by using the calibration line obtained from titration data. The recovery percentages for hydrazine detection were measured to be between 102.1% and 104.9% for tap water, between 94.8% and 107.6% for bottled water and between 94.7% and 111.1% for the lake samples (Table 1). This study demonstrated that hydrazine in real samples can reasonably be detected using **PHENOZ** as a fluorescent chemosensor.

**Table 1:** Quantification of N_2_H_4_ in different type of environmental water samples such as tap water of and bottled water

Type of water sample	Spiked N_2_H_4_ (µM)	Found N_2_H_4_ (µM) (*n* = 3)	Recovery (%)
Tap water	15.0	15.7 (± 0.1)	104.9
	20.0	20.9 (± 0.9)	104.2
	25.0	25.6 (± 0.4)	102.1
Bottled water	15.0	16.1 (± 0.1)	107.6
	20.0	18.9 (± 0.3)	94.8
	25.0	25.1 (± 0.3)	100.5
Lake water	15.0	14.2 (± 0.1)	94.7
	20.0	22.2 (± 2.4)	111.1
	25.0	24.8 (± 2.2)	99.2

### Characterization of PHENOZ-NNH_2_

**Scheme 2:**
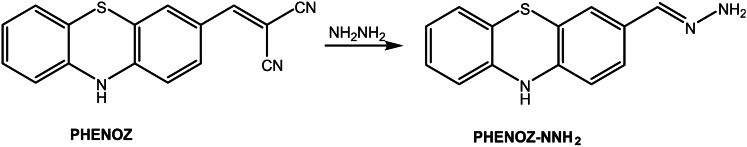
The proposed detection mechanism of sensor **PHENOZ** with hydrazine

Dicyanovinyl group is well known to be converted into hydrazone upon reaction with hydrazine (Scheme 2) [52, 61–63]. To prove that for **PHENOZ**, we isolated the product of the reaction of **PHENOZ** with hydrazine. The ^1^H NMR, ^13^C NMR, FTIR and HRMS spectra of the structure we obtained are consistent with the expected structure **PHENOZ**-**NNH₂** (Figs. S11-S15). In the **PHENOZ** sensor, the vinylic proton appears as a singlet at 8.05 ppm. Upon transformation to the **PHENOZ**-**NNH₂** structure, where a more electronegative nitrogen atom is incorporated into the molecule, this proton shifts downfield and is observed as a singlet at 8.45 ppm. This downfield shift of the vinylic proton provides strong evidence for the conversion of the **PHENOZ** structure into its corresponding hydrazone derivative.

When the FTIR spectra of the compounds were compared, a sharp band corresponding to the carbon-nitrogen triple bond stretching in the **PHENOZ** sensor was observed at 2220 cm⁻¹. The presence of this band was expected due to the dicyanovinyl moiety in the structure, which contributes characteristic cyanide stretching vibrations. In the case of the **PHENOZ-NNH₂** derivative, as anticipated, the stretching band attributed to the C ≡ N bond disappears. This absence is consistent with the transformation of the molecule into a hydrazone structure in the presence of hydrazine. The mass spectrum of this product was also in accordance with the structure.

This is attributed to a nucleophilic addition of hydrazine to the electrophilic β-carbon of the dicyanovinyl group, which disrupts the ICT pathway by reducing the electron-withdrawing capacity of the acceptor (Fig. S18). Consequently, the inhibition of ICT leads to the restoration of the locally excited (LE) emission from the phenothiazine core, resulting in a significant enhancement of fluorescence [77].

## Conclusion

In this study, we have developed and characterized a novel phenothiazine-based fluorescent probe **PHENOZ**. This compound was synthesized through a rapid and cost-effective two-step protocol using readily available starting materials, a distinct advantage over many previously reported multi-step hydrazine sensors that rely on expensive or synthetically challenging precursors. The molecular design integrates a phenothiazine fluorophore with a dicyanovinyl recognition unit, enabling hydrazine-specific reactivity through nucleophilic addition, which results in a marked turn-on fluorescence response.

A key finding of this work is the chemoselectivity of **PHENOZ**, which displays no detectable fluorescence response toward a broad panel of potentially interfering species, including cyanide and primary amines—two classes of nucleophiles that are known to react with dicyanovinyl-based probes. This unique selectivity fills a critical gap in the current chemosensor literature, where avoiding cross-reactivity with such species remains a major limitation. The low limit of detection 0.22 µM (7.05 ppb), falling well below the toxicity thresholds established by regulatory authorities such as ACGIH, underscores the analytical sensitivity of the system and confirms its suitability for trace-level monitoring of hydrazine.

The probe demonstrated good performance in environmental matrices, including tap water, bottled water, and lake water samples, with recovery values ranging from 94.7 to 111.1%, thereby affirming its reliability in real-world applications.

## Electronic Supplementary Material

Below is the link to the electronic supplementary material.

Supplementary Material 1

## Data Availability

No datasets were generated or analysed during the current study.
