# Supplementary material for: A Cost-effective Phenothiazine-based Fluorescent Chemosensor for Selective Detection of Hydrazine and its Application To Real Water Samples
Source: J Fluoresc. 2025 Jun 25;35(11):11815–24. doi: 10.1007/s10895-025-04421-9 (PMC12718247; doi:10.1007/s10895-025-04421-9)
Supplement: Supplementary file 1 — Supplementary Material 1 [file 10895_2025_4421_MOESM1_ESM.docx]

Electronic supplementary information for

**A Cost-effective Phenothiazine-based Fluorescent Chemosensor for Selective Detection of Hydrazine and its Application to Real Water Samples**

Özge Çağlar Teknikel^1,2*^ (0000-0001-6238-1302), Nebahat Değirmenbaşı^2*^ (0000-0002-2557-0160)

*^1^ Gazi University, Graduate School of Natural and Applied Sciences, 06500, Ankara/ Türkiye*
*^2^ Gazi University, Faculty of Science, Department of Chemistry, 06500, Ankara/ Türkiye*

^*^E-mail: [nebahatd@gazi.edu.tr](mailto:nebahatd@gazi.edu.tr) ; ozgeteknikel@gazi.edu.tr

**Contents**

Fig. S1 ^1^H NMR spectrum of **1** in DMSO 2

Fig. S2 Detailed ^1^H NMR spectrum of **1** in DMSO 3

Fig. S3 ^13^C NMR spectrum of **1** in DMSO 3

Fig. S4 FT-IR spectrum of **1** 4

Fig. S5 APCI-Mass spectrum of **1** 4

Fig. S6 ^1^H NMR spectrum of **PHENOZ** in DMSO 5

Fig. S7 Detailed ^1^H NMR spectrum of **PHENOZ** in DMSO 5

Fig.S8 ^13^C NMR spectrum of **PHENOZ** in DMSO 6

Fig. S9 FT-IR spectrum of **PHENOZ** 6

Fig. S10 APCI-Mass spectrum of **PHENOZ** 7

Fig. S11 ^1^H NMR spectrum of **PHENOZ**-**NNH_2_** 7

Fig. S12 Detailed ^1^H NMR spectrum of **PHENOZ**-**NNH_2_** 8

Fig. S13 ^13^C NMR spectrum of **PHENOZ**-**NNH_2_** 9

Fig. S14 FT-IR spectrum of **PHENOZ**-**NNH_2_** 9

Fig. S15 APCI-Mass spectrum of **PHENOZ-NNH_2_** 10

Fig. S16 Absorbtion spectrum of **PHENOZ** and **PHENOZ** upon addition of hydrazine 10

Fig. S17 Emision spectrum of **PHENOZ** and **PHENOZ-NNH_2_** 11

Fig. S18 Illustration of proposed sensing mechanism of **PHENOZ** 11

Table S1 Literature comparison of similar probes 12

References 13


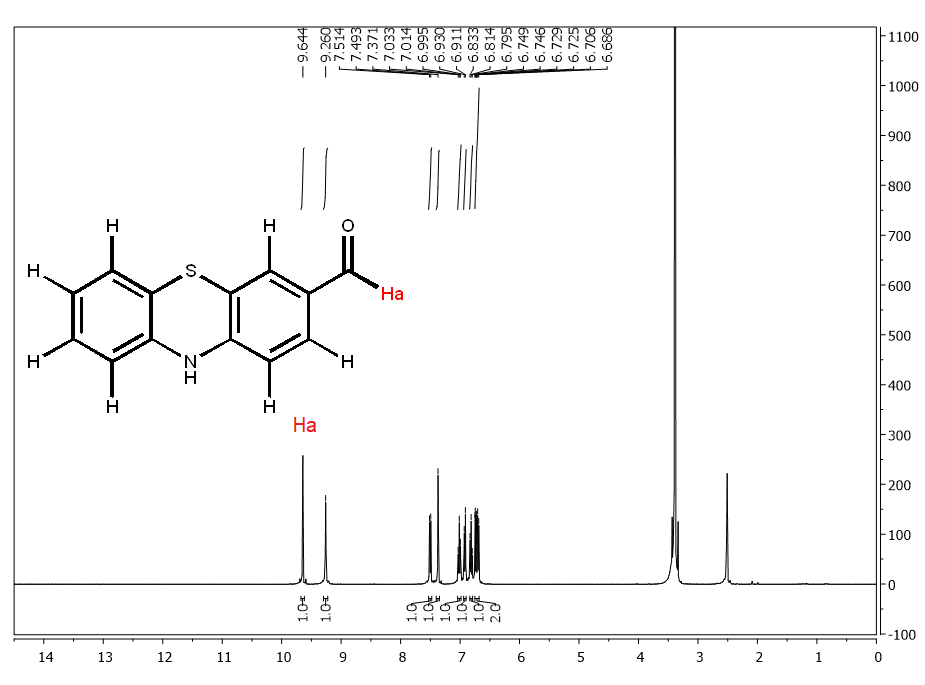


Fig. S1 ^1^H NMR spectrum of **1** in DMSO


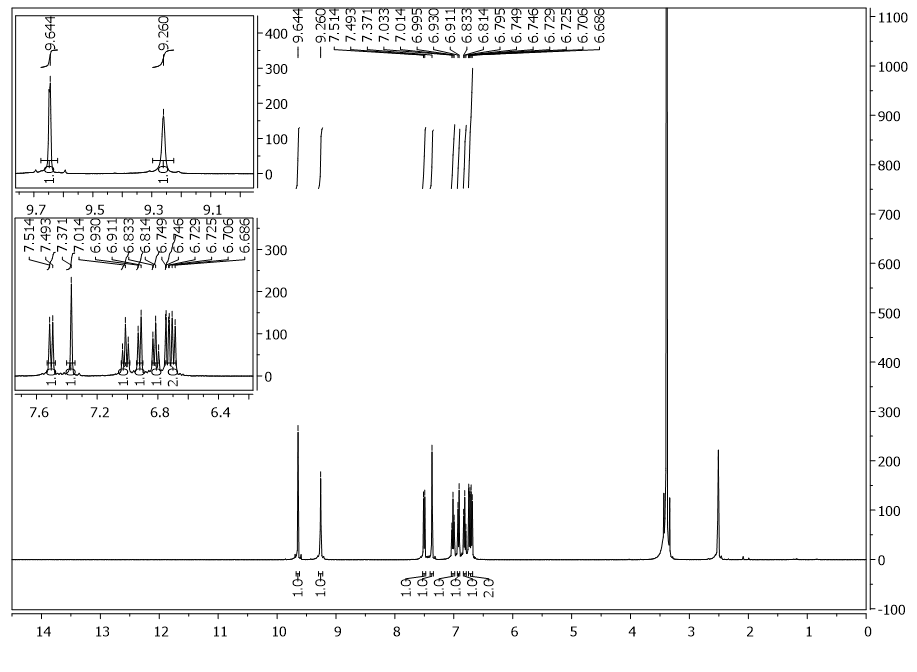


Fig. S2 Detailed ^1^H NMR spectrum of **1** in DMSO

**
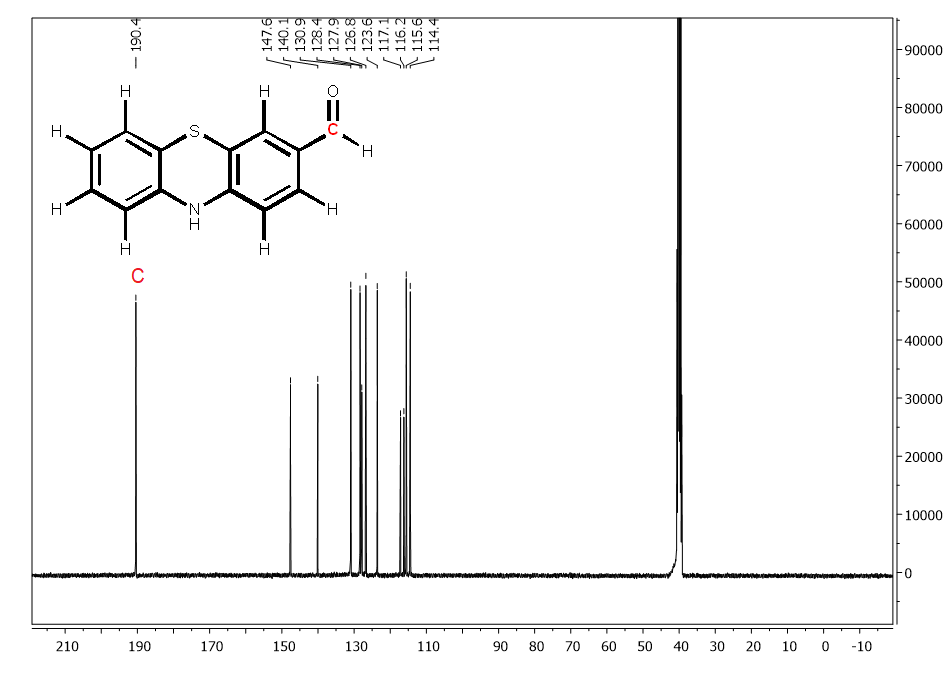
**

Fig. S3 ^13^C NMR spectrum of **1** in DMSO

Fig. S4 FT-IR spectrum of **1**

*
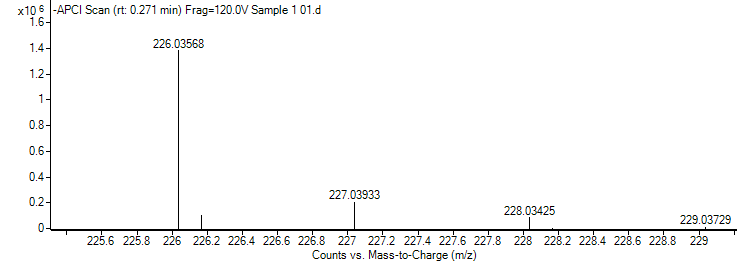
*

Fig. S5 APCI-Mass spectrum of **1**


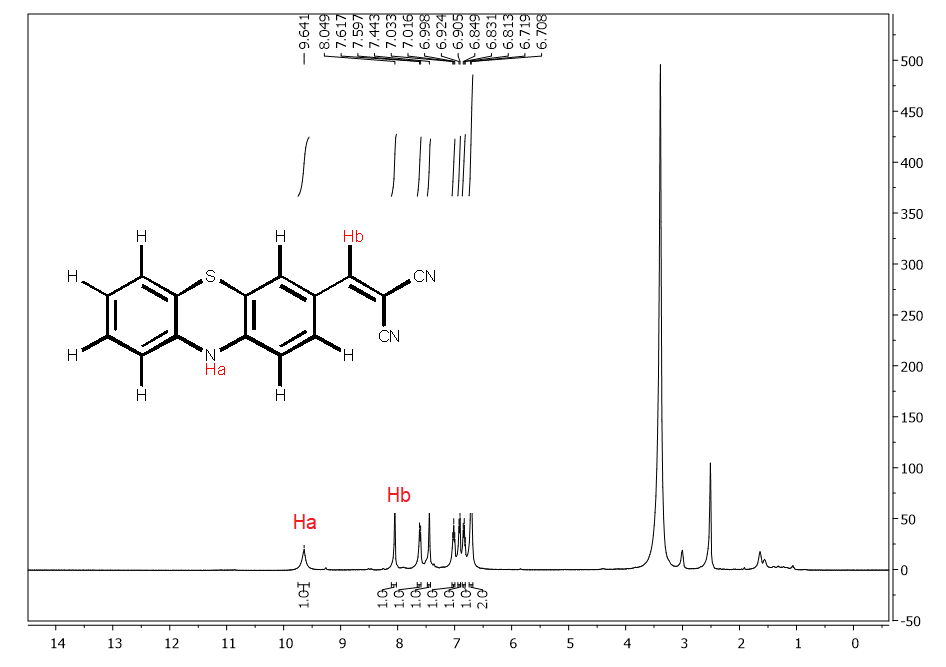


Fig. S6 ^1^H NMR spectrum of **PHENOZ** in DMSO


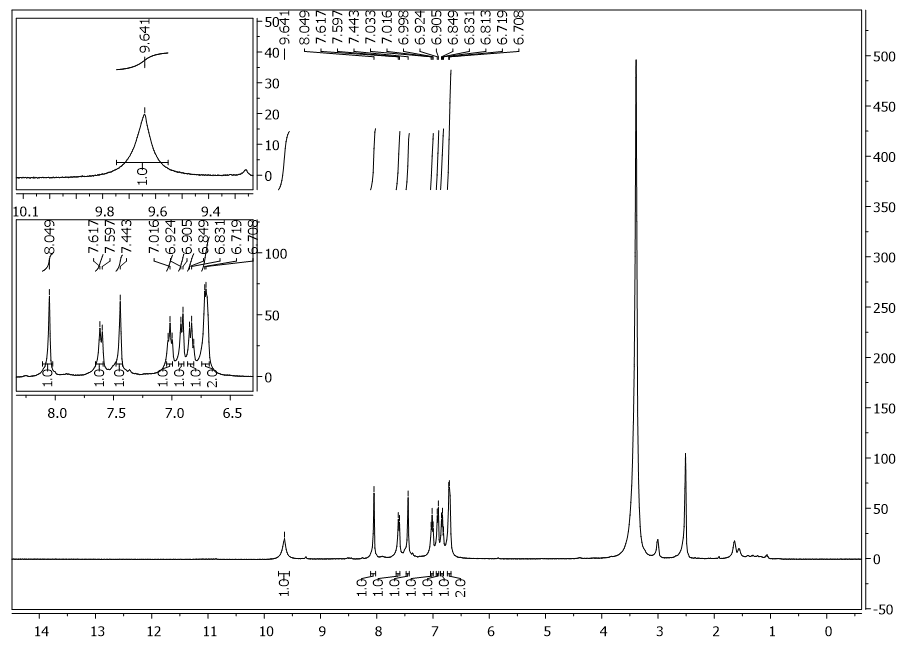


Fig. S7 Detailed ^1^H NMR spectrum of **PHENOZ** in DMSO


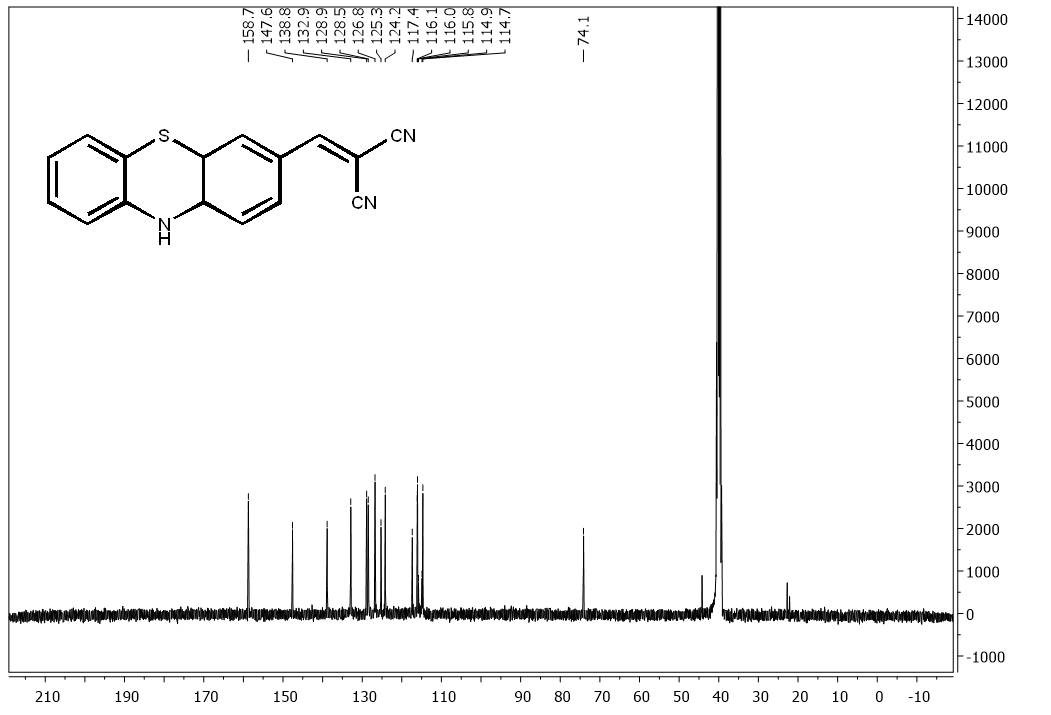


Fig. S8 ^13^C NMR spectrum of **PHENOZ** in DMSO

Fig. S9 FT-IR spectrum of **PHENOZ**


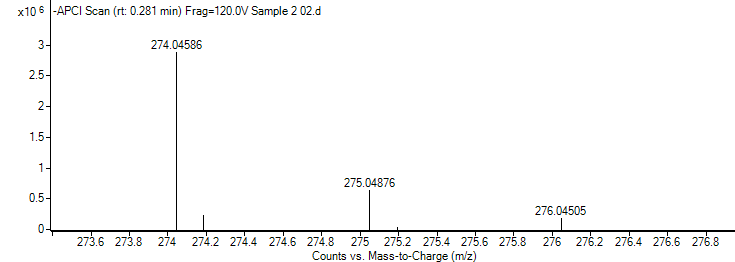


Fig. S10 APCI-Mass spectrum of **PHENOZ**


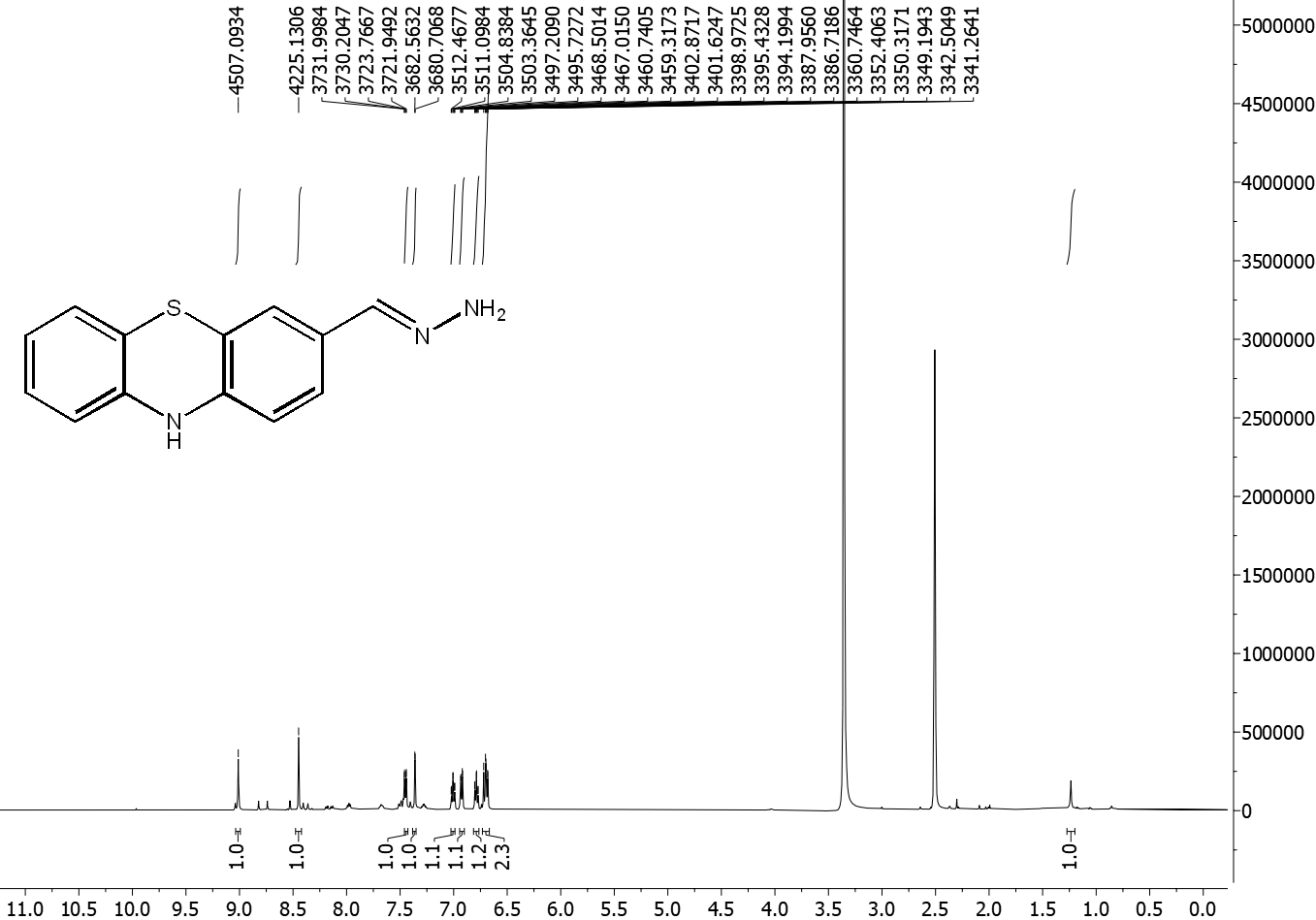


Fig. S11 ^1^H NMR spectrum of **PHENOZ-NNH_2_**

Fig. S12 Detailed ^1^H NMR spectrum of **PHENOZ-NNH_2_**


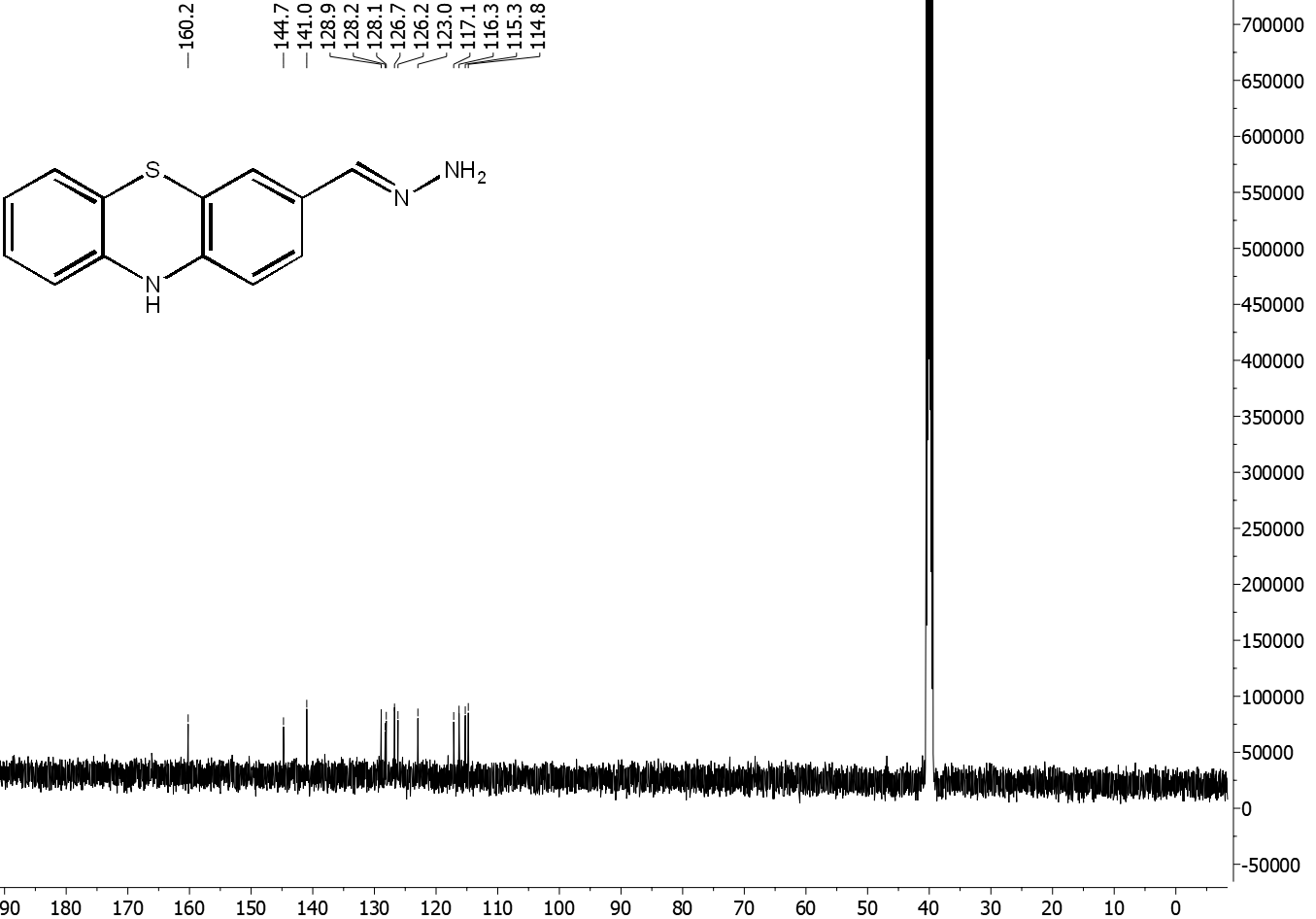


Fig. S13 ^13^C NMR spectrum of **PHENOZ-NNH_2_**

__

Fig. S14 FT-IR spectrum of **PHENOZ-NNH_2_**


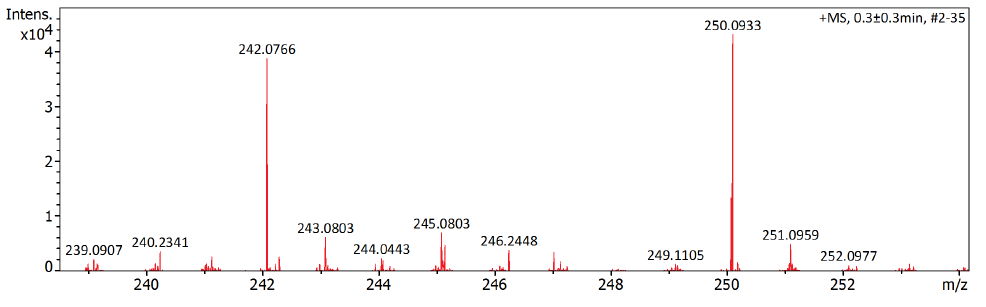


Fig. S15 HR-MS spectrum of **PHENOZ-NNH_2_**





Fig. S16 Absorbtion spectrum of **PHENOZ** and **PHENOZ** upon addition of hydrazine

Fig. S17 Emision spectrum of **PHENOZ** and **PHENOZ-NNH_2_**

Fig. S18 Illustration of proposed sensing mechanism of **PHENOZ**

Table S1: Literature comparison of similar probes

| No. | Probe | Solvent System | pH | Sensing  Responce | Analyte | λ_ex_/λ_em_ (nm) | LOD (µM) | Φ | Ref. |
| --- | --- | --- | --- | --- | --- | --- | --- | --- | --- |
| 1 |  | PBS/  EtOH:  Water (1:1) | 7.4 | Turn on | N_2_H_4_ and CN^-^ | 365/  540 | 0.08 | 0.62 | [1] |
| 2 |  | PBS/  EtOH:  Water (1:1) | 7.4 | Emission shift | N_2_H_4_ and  HSO_3_^-^ | 380/  490 | 0.01 | 0.54 | [2] |
| 3 |  | PBS/  EtOH:  Water (1:1) | 7.4 | Ratiometrical | N_2_H_4_ | 405/  540/  620 | 0.06  0.002 | 0.68 | [3] |
| 4 |  | EtOH | NA | Turn on | N_2_H_4_ | 380/  510 | 0.11 | 0.59 | [4] |
| 5 |  | CH_3_CN | NA | Chemodosimetric | N_2_H_4_ | NA/  NA | 0.0009 | NA | [5] |
| 6 |  | EtOH/  Water (1:1) | 7.4 | Ratiometric | N_2_H_4_ | 360/  475/  545 | 0.34 | NA | [6] |
| 7 |  | EtOH/  Water (7:3) | 7.4 | Turn on | N_2_H_4_ | 380/  525 | 0.19 | 0.57 | [7] |
| 8 |  | THF/  H_2_O (0.5:9.5) | pH < 5 | Turn on | N_2_H_4_ | 360/  510 | 0.105 | 0.1 | [8] |
| 9 |  | PBS/  EtOH:  Water (1:1) | 7.4 | Turn on | N_2_H_4_ | 400/  538 | 0.48 | 0.47 | [9] |
| 10 |  | EtOH: water  (9:1) | 7.4 | Turn on | N_2_H_4_ | 380/  500 | 0.22 | 0.05 | Our work |

References

1. Mu S, Gao H, Li C (2021) A dual-response fluorescent probe for detection and
bioimaging of hydrazine and cyanide with different fluorescence signals. Talanta
221:121606. <https://doi.org/10.1016/j.talanta.2020.121606>

2. Yang YZ, Qing M, Luo XY, et al (2022) A dual-response fluorescent probe for
discriminative sensing of hydrazine and bisulfite as well as intracellular imaging with
different emission. Spectrochim. Acta A Mol. Biomol. Spectrosc. 270:120795.
<https://doi.org/10.1016/j.saa.2021.120795>

3. Liu P, Wu WN, Wang Y, et al (2022) A dual-ratiometric mitochondria-targeted
fluorescent probe to detect hydrazine in soil samples and biological imaging.
J. Hazard. Mater. 440:129713. <https://doi.org/10.1016/j.jhazmat.2022.129713>

4. Jung MJ, Kim SJ, Lee MH (2020) π-Extended Tetraphenylethylene containing
a dicyanovinyl group as an ideal fluorescence turn-on and naked-eye color change probe
for hydrazine detection. *ACS Omega* 5:28369–28374.
<https://doi.org/10.1021/acsomega.0c04370>

5. Kasprowiak A, Rather IA, Ali R, Danjou PE (2023) Revisiting β-dicyanovinyl
substituted calix[4]pyrrole: Toward the chemodosimetric detection of hydrazine in
solution. J. Mol. Struct. 1287:135694. <https://doi.org/10.1016/j.molstruc.2023.135694>

6. Yi Q, He J, Fu X, Wang J, Tang Y, Liu L (2021) Carbazole-based chemosensor
for highly sensitive and selective bioimaging identification of hydrazine in multiple
model systems via ratiometric and colorimetric response. Dyes Pigments 196:109816.
<https://doi.org/10.1016/j.dyepig.2021.109816>

7. Wang Y, Yan Q, Wang Z, Xu H (2023) A flavonol-derived fluorescent probe for
highly specific and sensitive detection of hydrazine in actual environmental samples and
living zebrafish. Spectrochim. Acta A Mol. Biomol. Spectrosc. 288:122132.
<https://doi.org/10.1016/j.saa.2022.122132>

8. Rathore A, Kumar A, Nagrare K, Singh AK (2021) First fluorescence sensor for hydrazine ion: An effective “turn-on” detection based on thiophene-cyanodistyrene Schiff-base. J Fluoresc 31:647–657. https://doi.org/10.1007/s10895-021-02655-z

9. Xia HC, Wang HH, Han D et al (2024) Phenothiazine-based fluorescent probes
for the detection of hydrazine in environment and living cells. Talanta 269:125448.
<https://doi.org/10.1016/j.talanta.2023.125448>
